# Characterization of Cardiometabolic Risks in Different Combination of Anthropometric Parameters and Percentage Body Fat

**DOI:** 10.1038/s41598-019-50606-1

**Published:** 2019-10-01

**Authors:** Yuan-Yuei Chen, Wen-Hui Fang, Chung-Ching Wang, Tung-Wei Kao, Yaw-Wen Chang, Hui-Fang Yang, Chen-Jung Wu, Yu-Shan Sun, Wei-Liang Chen

**Affiliations:** 1 Department of Internal Medicine, Tri-Service General Hospital Songshan Branch, Taipei, Taiwan Republic of China; 2 Division of Family Medicine, Department of Family and Community Medicine, Tri-Service General Hospital; and School of Medicine, National Defense Medical Center, Taipei, Taiwan Republic of China; 3 Division of Geriatric Medicine, Department of Family and Community Medicine, Tri-Service General Hospital; and School of Medicine, National Defense Medical Center, Taipei, Taiwan Republic of China; 4 Graduate Institute of Clinical Medical, College of Medicine, National Taiwan University, Taipei, Taiwan Republic of China; 5 Division of Family Medicine, Department of Community Medicine, Taoyuan Armed Forces General Hospital, Taoyuan, Taiwan Republic of China; 6 Department of General Medicine, Tri-Service General Hospital, Taipei, Taiwan Republic of China

**Keywords:** Nutrition, Public health

## Abstract

The prevalence of obesity was increasing and became a growing problem worldwide. Obesity increased the risk of developing metabolic abnormalities and was associated adverse health outcomes. Our aim was to examine the associations among different combinations of obesity phenotypes (high body mass index > 27 kg/m^2^ (O), high waist circumference (male > 90 cm, female > 80 cm) (W), fatty liver (F) and percentage body fat in top 40% (P)) and cardiometabolic diseases (type 2 diabetes mellitus (DM), hypertension (HTN), metabolic syndrome (MetS)). A total of 48426 eligible subjects were categorized based on the different definitions. After adjusting for all covariables, participants with O + F + P combination were more likely associated with the presence of DM. Participants with O + W combination were more associated with the presence of HTN than others. Participants with O + W + F + P had higher risk for the presence of MetS than others. The study addressed the associations between different obesity phenotypes and DM and HTN in the adult population. Better understanding the pathophysiological mechanisms underlined individual vulnerability and progression of cardiometabolic insults.

## Introduction

The epidemiology of obesity was prevalent and increasing dramatically worldwide in the past three decades^1^. Recently, the trend of obesity was reported to be accelerating in some developed countries^2^. It had become a growing public health issue not only in kinds of comorbidities but also in quality of life in Taiwan^3^. Obesity was proposed to be related to several adverse health outcomes such as metabolic syndrome (MetS), diabetes mellitus (DM), hypertension (HTN) and increased morbidity and mortality^4^. Numerous studies had proposed the associations between different obesity indices with metabolic disorders. As a traditional and commonly used indicator, body mass index (BMI) was reported to be associated with MetS and the risks of DM^5^. Abdominal obesity, caused by visceral fat accumulation, was significantly associated with insulin resistance^6^. Waist circumference (WC) was considered as a better index to predict MetS than others^7^.

Body fat distribution was reported to have a significant association with the development of impaired glucose metabolism and MetS^8,9^. In a previous study, high percentage body fat (PBF) was associated with a high cardiometabolic risks in Korean population^10^. Besides, PBF had association with all-cause and cardiovascular mortality, high fat mass had higher mortality risk than BMI^11,12^. Therefore, it may be crucial to determine the clinical usefulness of measuring PBF to evaluate the obesity-associated metabolic dysfunction. The aim of our study was to detemine whether the different obesity phenotypes were closely related to glucose intolerance and HTN.

## Results

### Demographic characteristics of the study sample

All demographic information of study participants was shown in Table 1. The mean age of these obesity phenotypes was 43.7 ± 14.5, 44.9 ± 13.4, 48.7 ± 12.3, and 50.6 ± 13.4 years, respectively. All participants were divided into four obesity phenotypes: BMI > 27 kg/m^2^ (O), high waist circumference (male > 90 cm, female > 80 cm) (W), fatty liver (F), percentage body fat in top 40% (P). There were 1507, 3082, 3997, and 247 participants in O, W, F, and P phenotype, respectively.

**Table 1:** Characteristics of study sample in different obesity phenotypes.

Variables	BMI > 27 (O) (N = 1507)	High WC (W) (N = 3082)	Fatty liver (F) (N = 3997)	High PBF (P) (N = 247)
**Continuous Variables, mean (SD)**				
Age (years)	43.7 (14.5)	44.9 (13.4)	48.7 (12.3)	50.6 (13.4)
Body Mass Index (kg/m^2^)	30.1 (3.1)	28.1 (4.0)	25.8 (3.7)	27.9 (3.7)
Percentage body fat (%)	33.7 (6.7)	32.9 (6.5)	29.9 (7.0)	36.3 (5.0)
Waist circumference (cm)	93.5 (9.2)	92.4 (8.3)	87.6 (9.9)	92.4 (10.2)
Total cholesterol (mmol/L)	4.9 (0.9)	4.9 (0.9)	4.9 (0.9)	5.1 (0.9)
Uric acid (umol/L)	384.2 (90.4)	359.9 (90.4)	365.8 (89.2)	366.9 (91.0)
Creatinine (umol/L)	49.4 (17.0)	47.1 (17.6)	49.4 (15.3)	47.7 (17.6)
AST (units/L)	24.0 (13.5)	23.1 (13.3)	22.7 (12.1)	24.0 (13.9)
Albumin (g/L)	44.7 (3.0)	44.5 (3.0)	44.8 (2.7)	44.4 (2.7)
hsCRP (nmol/L)	31.4 (40.9)	26.7 (41.9)	24.8 (44.8)	30.5 (39.1)
**Category Variables, (%)**				
Gender (male)	6971 (65.6)	7179 (53.0)	6665 (67.7)	1973 (55.1)
Proteinuria	3047 (32.3)	3608 (31.4)	2659 (26.6)	861 (26.9)
Smoking	1641 (38.5)	2176 (31.4)	3570 (33.0)	1035 (29.0)
Drinking	2031 (55.2)	2895 (48.3)	4727 (49.4)	1440 (44.7)

### Association between obesity phenotypes and the presence of DM, HTN and MetS

Tables 2–4 showed the associations between different combinations of obesity phenotypes and the presence of DM, HTN, and MetS. There were 15 combinations as following: O, W, F, P, O + W, O + F, O + P, W + F, W + P, P + F, O + W + F, O + W + P, O + F + P, W + F + P, and O + W + F + P.

**Table 2:** Association between various combinations of obesity phenotypes and the presence of DM.

Variable	Model^a^ 1 OR (95% CI)	*P* Value	Model^a^ 2 OR (95% CI)	*P* Value	Model^a^ 3 OR (95% CI)	*P* Value
O (BMI > 27 kg/m^2^)	1.73 (0.23–13.01)	0.592	2.69 (0.35–20.51)	0.340	2.72 (0.36–20.83)	0.334
W (high waist circumference)	1.41 (0.72–2.78)	0.315	1.44 (0.72–2.87)	0.299	1.42 (0.71–2.84)	0.319
F (fatty liver)	3.81 (2.61–5.55)	<0.001	2.88 (1.95–4.25)	<0.001	2.85 (1.93–4.21)	<0.001
P (percentage body fat in top 40%)	3.38 (1.48–7.71)	0.004	1.92 (0.81–4.54)	0.138	1.93 (0.81–4.56)	0.136
O + W	2.68 (0.94–7.65)	0.067	3.38 (1.15–9.91)	0.027	3.34 (1.14–9.80)	0.028
O + F	2.22 (0.52–9.44)	0.280	2.08 (0.48–9.00)	0.327	2.11 (0.49–9.11)	0.318
O + P	—	—	—	—	—	—
W + F	4.26 (2.78–6.53)	<0.001	3.37 (2.17–5.24)	<0.001	3.31 (2.13–5.15)	<0.001
W + P	3.83 (1.75–8.36)	<0.001	2.36 (1.04–5.36)	0.040	2.32 (1.02–5.29)	0.045
P + F	4.16 (2.22–7.78)	<0.001	2.33 (1.20–4.51)	0.012	2.35 (1.21–4.57)	0.011
O + W + F	5.24 (3.20–8.58)	<0.001	4.61 (2.76–7.69)	<0.001	4.43 (2.65–7.40)	<0.001
O + W + P	7.10 (3.52–14.31)	<0.001	4.38 (2.08–9.20)	<0.001	4.25 (2.02–8.94)	<0.001
O + F + P	11.43 (4.78–27.32)	<0.001	8.31 (3.33–20.74)	<0.001	8.07 (3.23–20.16)	<0.001
W + F + P	6.26 (3.97–9.86)	<0.001	4.30 (2.66–6.96)	<0.001	4.19 (2.59–6.78)	<0.001
O + W + F + P	9.02 (6.22–13.08)	<0.001	6.63 (4.49–9.78)	<0.001	6.55 (4.44–9.67)	<0.001

^a^Adjusted covariates:

Model 1 = age.

Model 2 = Model 1 + proteinuria, serum total cholesterol, uric acid, creatinine, AST, albumin, hsCRP.

Model 3 = Model 2 + history of smoking, drinking.

**Table 3:** Association between various combinations of obesity phenotypes and the presence of HTN.

Variable	Model^a^ 1 OR (95% CI)	*P* Value	Model^a^ 2 OR (95% CI)	*P* Value	Model^a^ 3 OR (95% CI)	*P* Value
O (BMI > 27 kg/m^2^)	2.25 (1.01–5.05)	0.048	2.22 (0.98–5.04)	0.057	2.17 (0.96–4.93)	0.064
W (high waist circumference)	1.34 (1.01–1.78)	0.040	1.44 (1.07–1.93)	0.015	1.44 (1.07–1.93)	0.016
F (fatty liver)	1.66 (1.39–1.99)	<0.001	1.15 (0.95–1.39)	0.144	1.16 (0.96–1.40)	0.134
P (percentage body fat in top 40%)	2.30 (1.49–3.53)	<0.001	1.55 (0.99–2.43)	0.056	1.54 (0.98–2.42)	0.060
O + W	6.28 (4.05–9.74)	<0.001	5.35 (3.38–8.46)	<0.001	5.43 (3.43–8.60)	<0.001
O + F	3.73 (2.08–6.69)	<0.001	2.48 (1.36–4.51)	0.003	2.47 (1.35–4.50)	0.003
O + P	4.23 (1.00–17.78)	0.049	1.85 (0.41–8.42)	0.425	1.88 (0.41–8.58)	0.414
W + F	2.85 (2.32–3.51)	<0.001	2.25 (1.81–2.80)	<0.001	2.27 (1.83–2.83)	<0.001
W + P	2.15 (1.39–3.32)	<0.001	1.75 (1.11–2.76)	0.017	1.75 (1.11–2.76)	0.017
P + F	2.59 (1.85–3.62)	<0.001	1.53 (1.08–2.18)	0.018	1.52 (1.07–2.16)	0.021
O + W + F	3.20 (2.46–4.16)	<0.001	2.29 (1.74–3.01)	<0.001	2.33 (1.77–3.07)	<0.001
O + W + P	3.31 (2.12–5.16)	<0.001	2.40 (1.49–3.84)	<0.001	2.44 (1.52–3.91)	<0.001
O + F + P	2.91 (1.47–5.78)	0.002	1.60 (0.79–3.24)	0.189	1.64 (0.81–3.33)	0.167
W + F + P	3.43 (2.68–4.39)	<0.001	2.47 (1.89–3.22)	<0.001	2.49 (1.91–3.25)	<0.001
O + W + F + P	4.81 (3.98–5.80)	<0.001	3.36 (2.74–4.12)	<0.001	3.39 (2.77–4.16)	<0.001

^a^Adjusted covariates:

Model 1 = age.

Model 2 = Model 1 + proteinuria, serum total cholesterol, uric acid, creatinine, AST, albumin, hsCRP.

Model 3 = Model 2 + history of smoking, drinking.

**Table 4:** Association between various combinations of obesity phenotypes and the presence of MetS.

Variable	Model^a^ 1 OR (95% CI)	*P* Value	Model^a^ 2 OR (95% CI)	*P* Value	Model^a^ 3 OR (95% CI)	*P* Value
O (BMI > 27 kg/m^2^)	2.69 (0.80–8.99)	0.108	2.48 (0.74–8.39)	0.143	2.53 (0.75–8.59)	0.135
W (high waist circumference)	7.64 (5.57–10.48)	<0.001	8.62 (6.22–11.95)	<0.001	8.57 (6.18–11.88)	<0.001
F (fatty liver)	4.06 (3.11–5.32)	<0.001	2.94 (2.23–3.87)	<0.001	2.93 (2.22–3.86)	<0.001
P (percentage body fat in top 40%)	2.40 (1.21–4.76)	0.012	1.62 (0.81–3.26)	0.174	1.60 (0.80–3.22)	0.188
O + W	34.69 (21.50–55.96)	<0.001	28.16 (17.16–46.21)	<0.001	27.77 (16.90–45.62)	<0.001
O + F	5.63 (2.66–11.94)	<0.001	3.68 (1.72–7.91)	<0.001	3.74 (1.74–8.03)	<0.001
O + P	8.97 (1.78–45.11)	0.008	3.84 (0.72–20.55)	0.116	3.60 (0.66–19.54)	0.137
W + F	23.32 (17.48–30.48)	<0.001	20.23 (15.34–26.68)	<0.001	20.12 (15.25–26.54)	<0.001
W + P	13.78 (8.94–21.25)	<0.001	12.50 (7.94–19.68)	<0.001	12.36 (7.84–19.48)	<0.001
P + F	6.07 (3.99–9.24)	<0.001	3.74 (2.43–5.77)	<0.001	3.82 (2.47–5.89)	<0.001
O + W + F	47.19 (34.47–64.60)	<0.001	36.73 (26.59–50.75)	<0.001	36.25 (26.21–50.12)	<0.001
O + W + P	21.42 (13.55–33.86)	<0.001	16.14 (9.95–26.17)	<0.001	15.93 (9.80–25.88)	<0.001
O + F + P	17.93 (9.17–35.06)	<0.001	10.52 (5.29–20.93)	<0.001	10.65 (5.34–21.24)	<0.001
W + F + P	27.90 (20.70–37.60)	<0.001	21.82 (15.93–29.88)	<0.001	21.74 (15.87–29.78)	<0.001
O + W + F + P	51.34 (39.36–66.97)	<0.001	38.03 (28.91–50.04)	<0.001	38.26 (29.07–50.36)	<0.001

^a^Adjusted covariates:

Model 1 = age.

Model 2 = Model 1 + proteinuria, serum total cholesterol, uric acid, creatinine, AST, albumin, hsCRP.

Model 3 = Model 2 + history of smoking, drinking.

After fully adjusting for pertinent covariables, F was significantly associated with the presence of DM with odds ratios (ORs) of 2.85 (95% confidence interval (CI): 1.93–4.21). Other combinations of obesity phenotypes including O + W, W + F, W + P, P + F, O + W, O + W + P, O + F + P, W + F + P, and O + W + F + P also had significant association with the presence of DM (P < 0.05). O + F + P had risk for the presence of DM with ORs of 8.07 (95% CI: 3.23–20.16). W was associated with the presence of HTN with ORs of 1.44 (95% CI: 1.07–1.93). O + W, O + F, W + F, W + P, P + F, O + W + F, O + W + P, W + F + P, and O + W + F + P also had significant relationship with the presence of HTN. O + W had significantly higher risk of the presence of HTN with ORs of 5.43 (95% CI: 3.43–8.60) than others. W and F were associated with the presence of MetS with ORs of 8.57 (95% CI: 6.18–11.88) and 2.93 (95% CI: 2.22–3.86). O + W, O + F, W + F, W + P, P + F, O + W + F, O + W + P, O + F + P, W + F + P, and O + W + F + P were significantly associated with the presence of MetS (P < 0.05). O + W + F + P had higher risk for the presence of MetS than other combinations with odd ratios (ORs) of 38.26 (95% CI: 29.07–50.36).

## Discussion

In our study, the primary finding was that different obesity phenotypes were significantly associated with the presence of DM and HTN. The presence of DM was more likely associated with F phenotype than others. Those with W phenotype had closer relationship with HTN. Furthermore, subjects with different combinations of obesity phenotypes had higher risks of cardiometabolic diseases than those with single obesity phenotype. The combination of obesity phenotypes seems to have increased risk of obese individuals on the process for developing adverse health outcomes.

The clinical usefulness of PBF on MetS risks was addressed based on a nationally representative sample^13^. Cardiometabolic risk factors such as elevated blood pressure, dyslipidemia and hyperglycemia are substantially related to high PBF^10^. In two large scale population-based surveys, the high PBF group had higher risks of cardiovascular diseases than low PBF group among normal-weight males^14^. In an Italian study, subjects with high PBF had higher inflammatory biomarkers than control group, suggesting that high PBF might be an important indicator for MetS^15^. Obese subjects with higher PBF have higher insulin resistance and pro-inflammatory cytokines than those with normal BMI^16^. Insulin resistance plays an important role on the development of MetS caused by systemic inflammation with elevated circulating levels of CRP and TNF-α^17,18^.

Adiposity was a common risk factor for DM and cardiometabolic diseases^19^. Rodriguez demonstrated that body fat distribution was a strong influence on the development of glucose intolerance and DM^8^. Previous studies had addressed the important roles for abdominal adiposity and specifically visceral fat accumulation on the development of prediabetes and DM^6,20,21^. However, our findings revealed that general obesity was considered as a better phenotype for predicting the risks of DM for both gender groups. In a cross-sectional study of 4828 participants concerning the prediabetes and DM development, PBF may be more imperative than BMI and WC^22^. Shea *et al*. indicated that participants in the highest tertile of PBF had high risks of developing cardiometabolic disease compared to those with low PBF^23^. Excess body fat was associated with numerous comorbidities included HTN, insulin resistance, dyslipidemia and systemic inflammation. In patients with and without DM, the leptin-to-adiponectin ratio was a useful indicator to assess insulin resistance and atherosclerotic risks^24,25^.

Non-alcoholic fatty liver disease (NAFLD) was highly prevalent in patients with DM^26^. Numerous evidences had supported a strong relationship between NAFLD and diabetes risk that subjects would have approximately 5-fold risks of developing DM if they had NAFLD^27,28^. The presence of NAFLD and prediabetes or DM was associated with significant hepatic insulin resistance compared with subjects matched for adiposity without a fatty liver^29^. The plausible mechanism for NAFLD associated with prediabetes and DM in obesity was dysfunctional adipose tissue that promoted insulin resistance and pancreatic β-cell dysfunction^30,31^. Lopez and his colleagues demonstrated that the presence of insulin resistance of adipose tissue deteriorated the progression of glucose metabolism by multiple pathways that include subclinical inflammation and lipotoxicity^29^.

Increased WC, as defined as central obesity phenotypes in our study, was associated with elevated blood pressure^32^. Central type body fat distribution was more closely associated with renal-related hypertension than peripheral type obesity^33^. Previous studies had suggested that obesity-associated hypertension was characterized by an endothelial dysfunction^34^. The relationship between obesity and endothelial dysfunction might be through several pathways such as insulin resistance, inflammation and free fatty acid metabolisms^35^. Production of nitric oxide from oxidative stress might contribute to endothelial dysfunction in the pathophysiology of obesity. Hypoxia related adipose tissue inflammation secretes many reactive oxygen species and cytokines, leading to deterioration of nitric oxide signaling pathways in the endothelial cells^36^. Elevated endothelin-1 activity also played an important role in obesity- associated endothelial dysfunction^37^.

There were several potential limitations among our study. First, it was a cross-sectional design that casual inference was no assessible between obesity phenotypes and adverse outcomes. A longitudinal survey was suggested to be examined in further studies. Second, the study sample was obtained from health examinations in a single medical center. Limited ethnicity diversity in the participants might not reflect the association of obesity phenotypes and cardiometabolic diseases in racial difference. Third, the measurement for PBF in the health check-up was used by BIA, but not DEXA, a standard measurement for body composition with higher accuracy. Last, the diagnosis of fatty liver was determined by abdominal sonography, bias of image interpretation caused by physicians might occur. Despite the limitations mentioned above, our study had some advantages. A relatively large population-based sample included 20665 subjects was analyzed compared to others examined in small population. Besides, multivariable adjustment consisted with potential confounders and lifestyles such as smoking and alcoholic assumption was conducted in the statistical analysis.

## Conclusion

In summary, our study emphasized the important characteristics of these four obesity phenotypes and various combinations for different clinical implications. Identifying the distinct obesity phenotypes associated with DM and HTN in general population has implications for the stratification of cardiometabolic risk. Better understanding the pathophysiological mechanisms underlined individual vulnerability and progression of cardiometabolic insults. Therefore, association between body fat distribution with metabolic alternation and prolonged follow-up research for predicting risks of incident cardiometabolic diseases were necessary.

## Methods

### Study design

**Figure 1:**
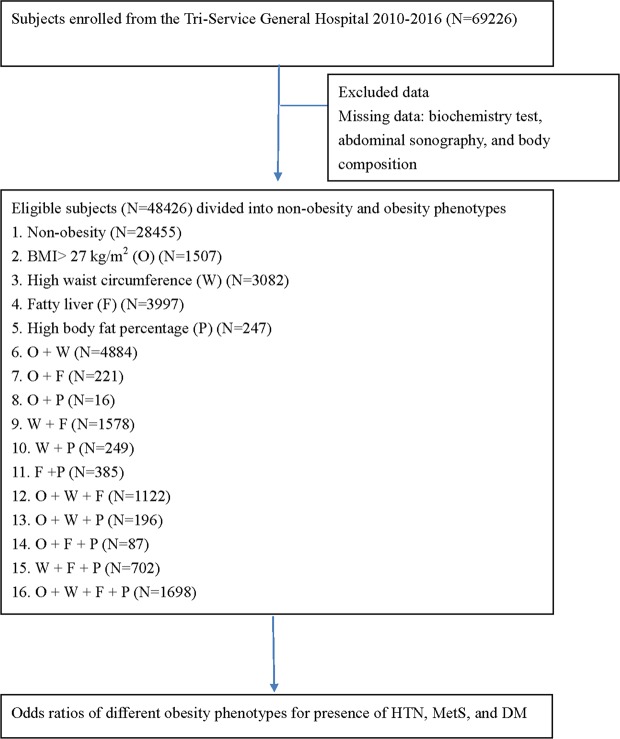
Flowchart of our study.

The retrospective cross-sectional was performed from health examinations in Tri-Service General Hospital from 2010 to 2016. There were 69226 participants received comprehensive examinations enrolling in the health check-up during the period. According to the step-by-step orders of flow chart shown in Fig. 1, subjects with missing data such as biochemistry data, body composition exams, abdominal sonography was excluded. 48426 eligible participants were divided into non-obesity and various obesity phenotypes as following: O was defined as a BMI > 27 kg/m^2^ according to the criteria of the Department of Health in Taiwan^4^; W was defined as subjects with high WC (male: > 90 cm; female > 80 cm); F was defined as subjects with fatty liver diagnosed by abdominal sonography. P was categorized into subjects along with PBF in top 40% of the entire obesity population.

The protocol was approved by the Tri-Service General Hospital Institutional Review Board based on the Declaration of Helsinki. Written informed consent was obtained from participants prior to the study. During the entire study process, characteristics of participants correlated with individual identification were eliminated and remained anonymously.

### Measurement of body composition

PBF was the indicator used in the study and was measured by BIA (InBody720, Biospace, Inc., Cerritos, CA, USA), an effective and validated method that was widely used for assessing body composition^38^.

### Definition of MetS

MetS was diagnosed when having ≥3 of the following features: (1) large waist circumference (WC): a waistline that measures at least 80 centimeters for women and 90 centimeters for men; (2) high triglyceride levels (TG): higher than 150 mg/dL; (3) reduced high-density lipoprotein cholesterol (HDL-C) levels: less than 50 mg/dL in women and 40 mg/dL in men; (4) increased blood pressure (BP): 130/85 mmHg or higher; and (5) elevated fasting plasma glucose (FPG): 100 mg/dL or higher based on the Harmonized criteria for MetS in 2009 with the Asian cut-off for WC^39,40^.

### Definition of type 2 DM

As reported by the American Diabetes Association criteria, subjects with one of the following components were diagnosed of T2DM: (1) fasting plasma glucose ≥126 mg/dL (2) hemoglobin A1c test ≥6.5% (3) random plasma glucose ≥200 mg/dL (4) past history of diabetes status, or use of antidiabetic agents^41^.

### Definition of HTN

Participants who had HTN was diagnosed by the guidelines of the Taiwan Society of Cardiology and the Taiwan Hypertension Society for the management of hypertension (1) blood pressure was higher than 140/90 mmHg (2) subjects were taking antihypertensive agents^42^.

### Covariates measurement

BMI was calculated by a formula that the weight of the subject in kilograms divided by the square of the height in meters (kg/m^2^). WC was measured at the mid-level between the iliac crest and the lower border of the 12^th^ rib. Biochemistry data was collected by drawing blood samples from subjects after fasting for at least 8 hours. Serum profiles included total cholesterol (TC), uric acid (UA), creatinine (Cr), aspartate aminotransferase (AST), albumin, and C-reactive protein (CRP) were analyzed by using standard methods.

### Statistical analysis

Statistical estimations used in the study were performed by the Statistical Package for the Social Sciences, version18.0 (SPSS Inc., Chicago, IL, USA) for Windows. The differences among these obesity phenotypes in terms of demographic information and biochemistry data were examined by analysis of variance and chi-square test. A two-sided *p*-value of ≤ 0.05 was regarded as the threshold for statistical significance. Extend-model approach was performed in the study with multivariable adjustment for pertinent clinical variables. ORs of different obesity phenotype combinations for the presence of DM and HTN were performed by a multivariate logistic regression.

## References

[1] James WP (2008). The epidemiology of obesity: the size of the problem. Journal of internal medicine.

[2] Rokholm B, Baker JL, Sorensen TI (2010). The levelling off of the obesity epidemic since the year 1999–a review of evidence and perspectives. Obesity reviews: an official journal of the International Association for the Study of Obesity.

[3] Chu NF (2005). Prevalence of obesity in Taiwan. Obesity reviews: an official journal of the International Association for the Study of Obesity.

[4] Hwang LC, Bai CH, Chen CJ (2006). Prevalence of obesity and metabolic syndrome in Taiwan. Journal of the Formosan Medical Association = Taiwan yi zhi.

[5] Ärnlöv J, Sundström J, Ingelsson E, Lind L (2011). Impact of BMI and the Metabolic Syndrome on the Risk of Diabetes in Middle-Aged Men. Diabetes Care.

[6] Usui C (2010). Visceral fat is a strong predictor of insulin resistance regardless of cardiorespiratory fitness in non-diabetic people. Journal of nutritional science and vitaminology.

[7] Shen W (2006). Waist Circumference Correlates with Metabolic Syndrome Indicators Better Than Percentage Fat. Obesity (Silver Spring, Md.).

[8] Rodriguez A, Catalan V, Gomez-Ambrosi J, Fruhbeck G (2007). Visceral and subcutaneous adiposity: are both potential therapeutic targets for tackling the metabolic syndrome?. Current pharmaceutical design.

[9] Kwon H, Kim D, Kim JS (2017). Body Fat Distribution and the Risk of Incident Metabolic Syndrome: A Longitudinal Cohort Study. Scientific reports.

[10] Kim JY, Han SH, Yang BM (2013). Implication of high-body-fat percentage on cardiometabolic risk in middle-aged, healthy, normal-weight adults. Obesity (Silver Spring, Md.).

[11] Lahmann PH, Lissner L, Gullberg B, Berglund G (2002). A prospective study of adiposity and all-cause mortality: the Malmo Diet and Cancer Study. Obesity research.

[12] Heitmann BL, Erikson H, Ellsinger BM, Mikkelsen KL, Larsson B (2000). Mortality associated with body fat, fat-free mass and body mass index among 60-year-old swedish men-a 22-year follow-up. The study of men born in 1913. International journal of obesity and related metabolic disorders: journal of the International Association for the Study of Obesity.

[13] Zhu S, Wang Z, Shen W, Heymsfield SB, Heshka S (2003). Percentage body fat ranges associated with metabolic syndrome risk: results based on the third National Health and Nutrition Examination Survey (1988–1994). The American journal of clinical nutrition.

[14] Tanaka S (2002). Is adiposity at normal body weight relevant for cardiovascular disease risk?. International journal of obesity and related metabolic disorders: journal of the International Association for the Study of Obesity.

[15] De Lorenzo A (2007). Normal-weight obese syndrome: early inflammation?. The American journal of clinical nutrition.

[16] Phillips CM (2013). Obesity and body fat classification in the metabolic syndrome: impact on cardiometabolic risk metabotype. Obesity (Silver Spring, Md.).

[17] Hu FB, Meigs JB, Li TY, Rifai N, Manson JE (2004). Inflammatory markers and risk of developing type 2 diabetes in women. Diabetes.

[18] Spranger J (2003). Inflammatory cytokines and the risk to develop type 2 diabetes: results of the prospective population-based European Prospective Investigation into Cancer and Nutrition (EPIC)-Potsdam Study. Diabetes.

[19] Grundy SM (2005). Diagnosis and management of the metabolic syndrome: an American Heart Association/National Heart, Lung, and Blood Institute Scientific Statement. Circulation.

[20] Neeland IJ (2012). Dysfunctional adiposity and the risk of prediabetes and type 2 diabetes in obese adults. Jama.

[21] Preis SR (2010). Abdominal subcutaneous and visceral adipose tissue and insulin resistance in the Framingham heart study. Obesity (Silver Spring, Md.).

[22] Gomez-Ambrosi J (2011). Body adiposity and type 2 diabetes: increased risk with a high body fat percentage even having a normal BMI. Obesity (Silver Spring, Md.).

[23] Shea JL, King MT, Yi Y, Gulliver W, Sun G (2012). Body fat percentage is associated with cardiometabolic dysregulation in BMI-defined normal weight subjects. Nutrition, metabolism, and cardiovascular diseases: NMCD.

[24] Inoue M, Maehata E, Yano M, Taniyama M, Suzuki S (2005). Correlation between the adiponectin-leptin ratio and parameters of insulin resistance in patients with type 2 diabetes. Metabolism: clinical and experimental.

[25] Inoue M, Yano M, Yamakado M, Maehata E, Suzuki S (2006). Relationship between the adiponectin-leptin ratio and parameters of insulin resistance in subjects without hyperglycemia. Metabolism: clinical and experimental.

[26] Gupte P (2004). Non-alcoholic steatohepatitis in type 2 diabetes mellitus. Journal of gastroenterology and hepatology.

[27] Shibata M, Kihara Y, Taguchi M, Tashiro M, Otsuki M (2007). Nonalcoholic fatty liver disease is a risk factor for type 2 diabetes in middle-aged Japanese men. Diabetes Care.

[28] Adams LA, Waters OR, Knuiman MW, Elliott RR, Olynyk JK (2009). NAFLD as a risk factor for the development of diabetes and the metabolic syndrome: an eleven-year follow-up study. The American journal of gastroenterology.

[29] Ortiz-Lopez C (2012). Prevalence of Prediabetes and Diabetes and Metabolic Profile of Patients With Nonalcoholic Fatty Liver Disease (NAFLD). Diabetes Care.

[30] Cusi K (2010). The role of adipose tissue and lipotoxicity in the pathogenesis of type 2 diabetes. Current diabetes reports.

[31] Kashyap S (2003). A sustained increase in plasma free fatty acids impairs insulin secretion in nondiabetic subjects genetically predisposed to develop type 2 diabetes. Diabetes.

[32] Siani A (2002). The relationship of waist circumference to blood pressure: the Olivetti Heart Study. American journal of hypertension.

[33] Scaglione R (1995). Central obesity and hypertension: pathophysiologic role of renal haemodynamics and function. International journal of obesity and related metabolic disorders: journal of the International Association for the Study of Obesity.

[34] Parrinello G (1996). Central obesity and hypertension: the role of plasma endothelin. American journal of hypertension.

[35] Prieto D, Contreras C, Sanchez A (2014). Endothelial dysfunction, obesity and insulin resistance. Current vascular pharmacology.

[36] Finkel T (2011). Signal transduction by reactive oxygen species. The Journal of Cell Biology.

[37] Sánchez A (2014). Endothelin-1 contributes to endothelial dysfunction and enhanced vasoconstriction through augmented superoxide production in penile arteries from insulin-resistant obese rats: role of ET(A) and ET(B) receptors. British Journal of Pharmacology.

[38] Roubenoff R (1996). Applications of bioelectrical impedance analysis for body composition to epidemiologic studies. The American journal of clinical nutrition.

[39] Alberti KG (2009). Harmonizing the metabolic syndrome: a joint interim statement of the International Diabetes Federation Task Force on Epidemiology and Prevention; National Heart, Lung, and Blood Institute; American Heart Association; World Heart Federation; International Atherosclerosis Society; and International Association for the Study of Obesity. Circulation.

[40] Tan CE, Ma S, Wai D, Chew SK, Tai ES (2004). Can we apply the National Cholesterol Education Program Adult Treatment Panel definition of the metabolic syndrome to Asians?. Diabetes Care.

[41] Tao Yuchun, Yu Jianxing, Tao Yuhui, Pang Hui, Yu Yang, Yu Yaqin, Jin Lina (2016). Comparison of the Combined Obesity Indices to Predict Cardiovascular Diseases Risk Factors and Metabolic Syndrome in Northeast China. International Journal of Environmental Research and Public Health.

[42] Chiang CE (2015). 2015 guidelines of the Taiwan Society of Cardiology and the Taiwan Hypertension Society for the management of hypertension. Journal of the Chinese Medical Association: JCMA.

